# Anakinra for Refractory Pseudogout in Patients with End-stage Renal Disease on Haemodialysis

**DOI:** 10.31138/mjr.261123.afr

**Published:** 2024-03-30

**Authors:** Christina Antoniadou, Nikolaos Fytanidis, Vasileios Devetzis, Konstantia Kantartzi, Charalampos Papagoras

**Affiliations:** 1 First Department of Internal Medicine, University Hospital of Alexandroupolis, Democritus University of Thrace, Alexandroupolis, Greece,; 2 “AKESIOS” Dialysis Centre, Alexandroupolis, Greece,; 3 Department of Nephrology, University Hospital of Alexandroupolis Democritus University of Thrace, Alexandroupolis, Greece

**Keywords:** calcium pyrophosphate deposition disease, pseudogout, end-stage renal disease, haemodialysis, interleukin-1β, anakinra

## Abstract

Calcium pyrophosphate deposition (CPPD) arthritis is the second most common type of crystal-induced arthritis after gout. Acute flares are commonly treated with non-steroidal anti-inflammatory drugs, intra-articular or short-term systemic glucocorticoids or colchicine. However, since there is no pharmacological treatment to reduce CPPD crystal burden, relapsing or chronic CPPD arthritis may be challenging to treat, particularly in patients with end-stage renal disease who are at risk for toxicity of the above medications. Since IL-1β appears to be driving CPPD arthritis, we treated two patients with chronic CPPD arthritis and end-stage renal disease on haemodialysis with the IL-1β receptor antagonist anakinra. In both patients, arthritis resolved quickly, while continuation of anakinra maintained remission and allowed complete glucocorticoid withdrawal. Therefore, anakinra may be a safe and effective option both for short and long-term treatment of CPPD arthritis in patients on chronic renal replacement therapy.

## INTRODUCTION

Crystal-induced arthritis is a well-known complication in patients with end-stage renal disease (ESRD) on renal replacement therapy. Except monosodium urate crystals, plenty of other calcium-containing crystals may deposit within joints and surrounding soft tissues, eliciting arthritis or tendinitis and often resulting in structural damage.^1,2^ Among non-gouty crystal-induced arthritides, calcium pyrophosphate deposition (CPPD) arthritis is the most frequent type in the general population.^3^ It is treated with short courses of non-steroidal anti-inflammatory drugs (NSAIDs) or glucocorticoids, while colchicine may be useful for long-term prophylaxis.^4^ However, when the underlying impairment of calcium metabolism persists, the disease may follow a chronic relapsing or refractory course,^1^ while treatment options are limited by short- or long-term drug toxicity, particularly in the background of ESRD. We describe 2 patients with ESRD on haemodialysis and pseudogout refractory to conventional treatments who responded to IL-1β blockade with anakinra.

## CASE DESCRIPTION

### Patient 1

A 62-year-old Caucasian male was referred to the outpatient rheumatology clinic for recurrent bouts of arthritis affecting primarily the small joints of the hands for the past 8 months. His joint symptoms had begun almost 10 months following the initiation of haemodialysis and usually remitted between flares.

His medical history included ESRD, possibly due to glomerulonephritis. A kidney biopsy, which provided a non-diagnostic tissue sample had been complicated with serious retroperitoneal haemorrhage and was not attempted again. Immunological tests, including antinuclear and anti-neutrophil cytoplasmic antibodies, were negative. Despite treatment with glucocorticoids and immunosuppressants, the patient gradually progressed to ESRD requiring haemodialysis three times weekly. His chronic medications included methylprednisolone (4mg/day), furosemide, amlodipine, terazosin, allopurinol, sevelamer, epoetin, bemiparin, atorvastatin, folate, and omeprazole.

When evaluated during a flare of the arthritis, he had asymmetric joint involvement with swelling, warmth, and tenderness of the wrist, the first metacarpophalangeal and the third proximal and distal interphalangeal joints of the left hand. No fever or other abnormal findings could be noted.

**Figure 1.**
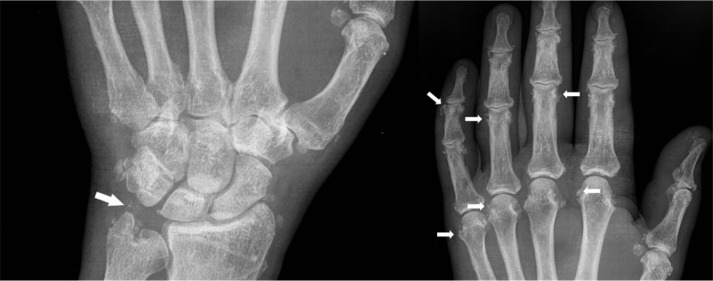
Hand radiographs of patient 1. Left: Chondrocalcinosis of the triangular fibrocartilage of the wrist (arrow). Right: Soft tissue calcifications of the finger joints (arrows).

Laboratory tests showed normal white blood cell count and a mild elevation of C-reactive protein (2.77mg/dL). Serum urate was 5.8mg/dL, calcium 10.2mg/dL, phosphorus 6.1mg/dL and parathyroid hormone (PTH) 218.3pg/mL, the latter being within target range considering the presence of ESRD. Rheumatoid factor (RF) and anti-cyclic citrullinated peptide (anti-CCP) antibodies were negative. Small joint aspiration failed to provide synovial fluid for microscopic examination, while hand X-rays revealed chondrocalcinosis of the triangular cartilage of the wrist and calcifications in the periarticular soft tissues and hooklike osteophytes, suggestive of CPPD (**Figure 1**).

The differential diagnosis included CPPD-arthritis, gout, and rheumatoid arthritis. Gout was considered less likely, as the patient had never had significant hyperuricemia before or over the course of the arthritis. Furthermore, the pattern of arthritis (attacks of asymmetrical oligoarthritis with symptom-free intervals) was not consistent with rheumatoid arthritis. A diagnosis of CPPD was thus made on the grounds of clinical presentation and radiographic findings.

Initial treatment included colchicine which was increased to the highest tolerated dosage (0.5mg/day), hydroxychloroquine (200mg b.i.d.) and low-dose methylprednisolone (4–8mg/day). However, the arthritic flares gradually worsened, and the disease transitioned to chronic poly-arthritis of the upper extremities. Moreover, long-term glucocorticoid use resulted in osteoporosis and a mild cushingoid syndrome. Considering that CPPD arthritis may respond to IL-1β blockade,^5^ we administered a sub-cutaneous injection of anakinra 100mg 3 times weekly at the end of each haemodialysis session for 5 consecutive sessions, which resulted in a rapid resolution of the arthritis. However, one week after the last anakinra injection the arthritis relapsed, which prompted us to resume anakinra at the same dosage. With this treatment the patient remained symptom-free over the next 6 months. The drug was well tolerated, while hydroxychloroquine, colchicine and glucocorticoids could be completely withdrawn. Subsequently, anakinra was tapered to twice and then once per week, until it was discontinued after a few months due to sustained remission of the arthritis. Following the discontinuation, the patient remained symptom-free for the last 2 years.

### Patient 2

A 54-year-old Caucasian male was referred for arthritis of his left shoulder. It had begun almost one year ago with recurring flares of arthritis, it was often accompanied by low-grade fever, and gradually evolved into chronic monoarthritis of the left shoulder. His medical history included diabetes mellitus for 20 years, which was the cause for his ESRD requiring haemodialysis during the last 2 years. He had no history of psoriasis, inflammatory bowel disease or another rheumatic disorder. His long-term medications included candesartan, amlodipine, furosemide, carvedilol, low dose aspirin, insulin, calcium supplementation, epoetin and, until recently, sucroferric oxyhydroxide. On clinical examination there was obvious effusion of the left glenohumeral joint, which was warm, tender and had a limited range of motion. Laboratory evaluation showed increased C-reactive protein (12mg/dL) and PTH (176 pg/mL), while serum calcium, magnesium, phosphorus, urate and TSH were normal. Upon aspiration, synovial fluid was slightly haemorrhagic with pleiocytosis (29,000 leukocytes/μL, 97% neutrophils), but no crystals could be clearly recognised on light microscopy. Synovial fluid culture and a polymerase chain reaction assay for mycobacterium tuberculosis were also negative. Radiographs showed no calcium deposition in musculoskeletal structures, while magnetic resonance imaging of the shoulder showed glenohumeral joint effusion with synovial hypertrophy, as well as an abundant effusion in the subacromial bursa and around the tendon of the long head of the biceps.

Since septic arthritis and gout were excluded, there was no evidence suggestive of peripheral spondyloarthritis, and considering the pattern of the arthritis (self-limiting bouts of monoarthritis accompanied by fever, gradually evolving into a chronic arthritis), CPPD arthritis appeared as the most plausible diagnosis.^7^ Although the identification of CPP crystals in the synovial fluid or tissue confirms the diagnosis, small rhomboid crystals are often difficult to detect. As the patient declined intra-articular injection of glucocorticoids, we prescribed oral methylprednisolone 16mg/day combined with colchicine up to 0.5mg/day, which produced an improvement over the next few days. However, repeated attempts to taper methylprednisolone to 8mg/day resulted in arthritis relapse despite background colchicine. Thus, we initiated anakinra 100mg 3 times weekly after each haemodialysis session, to spare glucocorticoids. With anakinra residual inflammation subsided quickly, while the joint effusion and synovial hyper-trophy regressed completely over the following weeks and the joint regained its full range of motion. After 6 months, anakinra was tapered to twice per week with sustained remission over another 3 months of follow up. No adverse events were reported by the patient.

## DISCUSSION

Calcium pyrophosphate deposition disease is considered the third most common cause of inflammatory arthritis and the most common cause of chondrocalcinosis.^6–8^ Several phenotypes are associated with CPPD including asymptomatic CPPD, osteoarthritis with CPPD, acute CPPD arthritis, and chronic CPPD arthritis.^7^ Although the pathogenesis of the disease is not fully understood, the initiating event appears to be the formation of CPP crystals in the pericellular cartilage matrix.^3^ The subsequent activation of the components of NLRP3 inflammasome, leading to the production of mature interleukin-1β (IL-1β), is essential to trigger the inflammation.^3, 9–10^

A major risk factor for CPPD disease is age, as it is quite rare in people younger than 60 years old.^3,11–12^ Other risk factors include osteoarthritis,^6,11,13–14^ previous joint trauma^15^ and disorders of calcium metabolism, such as hyperparathyroidism, hypophosphatasia, and hypomagnesemia.^3^ Chronic kidney failure is associated with a 2.3-fold higher risk for pseudogout,^16^ while the prevalence of a CPPD-pattern in patients on haemodialysis and joint disease has been reported as high as 43%.^17^

Chronic kidney disease has been associated with several other types of calcium-containing crystal deposition, such as basic calcium phosphate (BCP), hydroxyapatite and calcium oxalate crystals.^1^ Deposition of BCP crystals usually affects the shoulder causing calcific tendinitis/periarthritis or, when present intra-articularly, a destructive form of arthropathy called the Milwaukee shoulder.^18^ Hydroxyapatite crystals are usually found at periarticular sites and are a cause of periarthritis rather than frank arthritis.^19^ Oxalate is a metabolic end-product that is normally excreted in the urine, and also removed by haemodialysis. Accumulation of oxalate in patients on renal replacement therapy occurs infrequently and may result in the deposition of calcium oxalate crystals and musculoskeletal manifestations resembling CPPD.^1–2, 20^ Although arthritis due to the above conditions is much rarer than CPPD, given the clinical overlap and radiographic similarities among them, the microscopic identification of the crystals, occasionally using special stains in the synovial fluid or tissue samples, is the most accurate method for definite diagnosis.^1^ However, apart from technically challenging, this may be clinically redundant, given that the mechanisms of inflammation likely converge to IL-1β production.^18, 21–22^ On the other hand, synovial fluid aspiration is most important to rule out infectious arthritis due to Gram-positive cocci or even mycobacteria. Indeed, septic arthritis, particularly if a single joint is involved, is the most clinically relevant differential diagnosis,^7^ given that haemodialysis patients are at greater risk due to ESRD-related immune dysfunction and repeated vascular access and considering the hazards of treating such a condition with immune suppressing medications.^23^

Regarding treatment, most evidence concerns CPPD disease. Acute attacks are managed with NSAIDs, intra-articular glucocorticoids, colchicine, or a short course of systemic glucocorticoids. In contrast with hyperuricemia and gout, there are no pharmacologic treatments to remove the CPP crystal burden. Therefore, chronic CPPD arthritis may be less responsive to long-term anti-inflammatory treatment with colchicine, low-dose NSAIDs or glucocorticoids, while methotrexate or hydroxychloroquine have also been recommended.^4^ However, in the setting of ESRD, some of those drugs are contraindicated (NSAIDs, methotrexate) or associated with significant toxicity (colchicine, glucocorticoids).

A breakthrough in the understanding of crystal-associated arthritides has been the recognition of the role of inflammasome and IL-1β in mediating crystal-induced inflammation.^24^ Indeed, anti-IL-1 therapies, such as the recombinant IL-1 receptor antagonist anakinra, have been effective for the treatment of refractory gout.^25^ Regarding CPPD arthritis there are only a few case reports, case series and observational studies^5,26–33^ supporting the use of anakinra. Most studies have assessed its short-term efficacy in acute CPPD disease, while there are limited data about its long-term use for chronic CPPD arthritis or for prophylaxis.^28, 34^ Overall, most patients in the above studies, showed a good or partial response and the drug was well tolerated.^35^ Relapses occurred in 27.3–37.5% of patients within a few months of drug discontinuation.^26,29,34–35^ Regarding concomitant ESRD, there is a single case report of a patient with CPPD arthritis who responded favourably to anakinra.^33^ From a pharmacological point of view, the drug is eliminated mainly by renal excretion and in haemodialysis patients it is advised to administer every other day.^36^ We thus prescribed it on a thrice-weekly basis and on close monitoring, which was efficacious with no adverse events in the short and medium term. Following sustained remission, the drug was slowly tapered and discontinued completely in one of the patients with no relapse over the following 2 years. Apparently, if the disease relapses, re-introduction of anakinra would most likely be the treatment of choice. Besides anakinra, to date there are no reports assessing the efficacy of other interleukin-1β inhibitors, such as canakinumab, in CPPD arthritis. For this reason, as well as due to lower costs we chose anakinra for both of our patients. However, in case of intolerance to anakinra (eg, due to injection site reactions) or loss of efficacy, canakinumab could be the next of choice based on the mechanism of action.

## CONCLUSION

In conclusion, both cases presented here suggest that anakinra is effective and safe for the rapid resolution of CPPD arthritis, as well as for long-term prophylactic therapy in patients with ESRD on haemodialysis. The drug was well tolerated and could be tapered and eventually discontinued following several months of sustained remission. Large clinical trials should be undertaken to formally assess the role of IL-1 inhibitors in CPPD disease, particularly in patients with ESRD who have fewer therapeutic alternatives.

## ABBREVIATIONS

CCP: Cyclic citrullinated peptide
BCP: Basic calcium phosphate
CPPD: Calcium pyrophosphate deposition
ESRD: end-stage renal disease
IL: interleukin
NSAIDs: non-steroidal anti-inflammatory drugs
PTH: Parathyroid hormone
RF: Rheumatoid factor
